# 4119 Cholecystokinin (CCK) Receptor Antagonist Reverses Nonalcoholic Steatohepatitis (NASH) by Reducing Hepatic Macrophages and Inflammatory Cytokines

**DOI:** 10.1017/cts.2020.293

**Published:** 2020-07-29

**Authors:** Martha Gay, Anita Safronenka, Hong Cao, Robin Tucker, Narayan Shivapurkar, Annie Kruger, Jill Smith

**Affiliations:** 1 Georgetown - Howard Universities; 2 Georgetown University

## Abstract

OBJECTIVES/GOALS: NASH increases the risk of cirrhosis and liver cancer. High-fat diets increase CCK levels and CCK receptors have been identified on fibroblasts and immune cells. We hypothesized that CCK receptor blockade could prevent NASH by altering the hepatic microenvironment and macrophage activation. METHODS/STUDY POPULATION: Female mice were fed a Choline Deficient Ethionine supplemented (CDE) saturated fat diet or control high-fat diet for 18 weeks. Mice in each group were treated with a CCK receptor antagonist, proglumide (0.1 mg/ml) in the drinking water or regular water. Resected livers were stained for H&E for features of NASH and F4/80 for macrophages analysis. Liver RNA was evaluated for the expression of cytokines and chemokines using an 84-gene Profiler array (Qiagen). Oxidative stress was analyzed by qRT-PCR for heat shock proteins (HSPs) 27, 60, 70 and 90 and for glutathione by a fluorometric assay. Differences in CDE fed and CDE/proglumide-treated mouse livers were evaluated. RESULTS/ANTICIPATED RESULTS: Livers from mice on the CDE diet displayed histologic features of NASH that were prevented by proglumide. Cytokines and chemokines expression, especially CCL20 and CCL2, were increased in the CDE fed mice and these levels were reduced greater than 20-fold with proglumide. Infiltration of F4/80+ macrophages was markedly increased in the CDE livers and these were reduced by > 50% (p < 0.0001) with proglumide. RNA expression of HSP70 (p = 0.006) and HSP27 (p = 0.011) were reduced with proglumide. Hepatic glutathione concentration more than doubled in the CDE/proglumide treated mice compared to CDE mice. CCK-B receptor expression increased in the CDE-fed mouse livers compared to controls. DISCUSSION/SIGNIFICANCE OF IMPACT: CCK receptor blockade decreases NASH by reducing hepatic macrophages, oxidative stress, and blocking inflammatory cytokines and chemokines. This data supports our novel hypothesis that CCK receptors play a role in NASH and proglumide may provide an innovative treatment for this condition.

